# National, State-Level, and County-Level Prevalence Estimates of Adults Aged ≥18 Years Self-Reporting a Lifetime Diagnosis of Depression — United States, 2020

**DOI:** 10.15585/mmwr.mm7224a1

**Published:** 2023-06-16

**Authors:** Benjamin Lee, Yan Wang, Susan A. Carlson, Kurt J. Greenlund, Hua Lu, Yong Liu, Janet B. Croft, Paul I. Eke, Machell Town, Craig W. Thomas

**Affiliations:** ^1^Division of Population Health, National Center for Chronic Disease Prevention and Health Promotion, CDC; ^2^Oak Ridge Institute for Science and Education, Oak Ridge, Tennessee.

Depression is a major contributor to mortality, morbidity, disability, and economic costs in the United States (*1*). Examining the geographic distribution of depression at the state and county levels can help guide state- and local-level efforts to prevent, treat, and manage depression. CDC analyzed 2020 Behavioral Risk Factor Surveillance System (BRFSS) data to estimate the national, state-level, and county-level prevalence of U.S. adults aged ≥18 years self-reporting a lifetime diagnosis of depression (referred to as depression). During 2020, the age-standardized prevalence of depression among adults was 18.5%. Among states, the age-standardized prevalence of depression ranged from 12.7% to 27.5% (median = 19.9%); most of the states with the highest prevalence were in the Appalachian* and southern Mississippi Valley^†^ regions. Among 3,143 counties, the model-based age-standardized prevalence of depression ranged from 10.7% to 31.9% (median = 21.8%); most of the counties with the highest prevalence were in the Appalachian region, the southern Mississippi Valley region, and Missouri, Oklahoma, and Washington. These data can help decision-makers prioritize health planning and interventions in areas with the largest gaps or inequities, which could include implementation of evidence-based interventions and practices such as those recommended by The Guide to Community Preventive Services Task Force (CPSTF) and the Substance Abuse and Mental Health Services Administration (SAMHSA).

BRFSS is an ongoing, state-based, random-digit–dialed landline and cell phone survey of the U.S. adult population aged ≥18 years in all 50 states, the District of Columbia (DC), and participating U.S. territories.^§^ The combined (landline and cellular) median response rate for the 2020 BRFSS (excluding territories) was 47.6% and ranged among states from 34.5% to 67.2%.^¶^ A lifetime diagnosis of depression was defined as a “yes” response to the question, “Has a doctor, nurse, or other health professional ever told you that you had a depressive disorder, including depression, major depression, dysthymia, or minor depression?” Among the 2020 BRFSS respondents surveyed in all 50 U.S. states and DC, 392,746 (98.9%) responded to the depression question.

This report presents national, state-level, and county-level point estimates and 95% CIs for the prevalence of depression. National values were directly estimated from weighted BRFSS 2020 data for groups defined by age, sex, race or ethnicity, and education, and state-level estimates were directly estimated from weighted BRFSS 2020 data for each state and DC. Point estimates from survey data were estimated as weighted means and pairwise t-tests were used to determine differences (compared with a reference category) by age group, sex, race or ethnicity, and education level. Differences with p<0.05 were considered statistically significant. Because BRFSS is not designed to provide estimates at the county level, county-level estimates were obtained for all 3,143 U.S. counties using multilevel logistic regression and post-stratification.** The multilevel logistic regression model included depression as the binary dependent variable. The model’s independent variables included each respondent’s age group, sex, race and ethnicity, and education level from BRFSS 2020 data, county-level poverty data (<150% of the poverty level) from the 2016–2020 American Community Survey,^††^ and random effects for state and county. The model parameters were then applied to the U.S. Census Bureau Vintage 2020 county population data to generate model-based county-level estimates of depression prevalence.^§§^ A Monte Carlo simulation was used to generate 95% CIs for county-level estimates. These model-based county-level estimates were validated by comparing them with the weighted direct survey estimates from counties with sample size ≥500 (183) in BRFSS (Pearson correlation coefficient = 0.88). All national and state-level analyses were conducted using SAS-callable SUDAAN software (version 11; RTI International) to account for the BRFSS complex sample design and weighting, and county-level estimation was conducted using SAS software (version 9.4; SAS Institute). All prevalence estimates were age standardized to the 2000 U.S. Census Bureau population.^¶¶^ This activity was reviewed by CDC and was conducted consistent with applicable federal law and CDC policy.***

The age-standardized prevalence of depression among U.S. adults was 18.5% (crude = 18.4%) (Table 1). Age-specific prevalence of depression was highest among those aged 18–24 years (21.5%) and lowest among those aged ≥65 years (14.2%). The age-standardized prevalence of depression was higher among women (24.0%) compared with men (13.3%), higher among non-Hispanic White adults (21.9%) compared with non-Hispanic Black or African-American (16.2%), non-Hispanic Native Hawaiian or other Pacific Islander (14.6%), Hispanic or Latino (14.6%), and non-Hispanic Asian (7.3%) adults, and higher among adults who had attained less than a high school education (21.2%) compared with adults with a high school education or equivalent (18.5%) and college degree or higher (15.4%).

**TABLE 1:** Prevalence estimates of adults aged ≥18 years self-reporting a lifetime diagnosis of depression,* by selected characteristics — Behavioral Risk Factor Surveillance System, United States, 2020

Characteristic	Sample size^†^	Unweighted no. with depression^§^	Weighted^§^ no. with depression (millions)	Prevalence^§^ % (95% CI)	Age-standardized prevalence^¶^ % (95% CI)
**Overall**	**392,746**	**74,830**	**47.0**	**18.4 (18.1–18.6)**	**18.5 (18.2–18.8)**
**Age group, yrs**					
18–24 (Ref)	24,850	5,688	6.6	21.5 (20.6–22.5)	NA
25–44	93,614	20,862	17.2	19.9 (19.5–20.4)**	NA
45–64	136,704	27,876	15.2	18.4 (17.9–18.9)**	NA
≥65	137,578	20,404	8.0	14.2 (13.7–14.6)**	NA
**Sex**					
Men (Ref)	179,937	24,587	16.3	13.1 (12.8–13.4)	13.3 (12.9–13.6)
Women	212,809	50,243	30.7	23.4 (22.9–23.8)**	24.0 (23.6–24.5)**
**Race and ethnicity**					
AI/AN, non-Hispanic	6,887	1,455	0.6	23.3 (21.1–25.7)**	23.4 (21.1–25.9)
Asian, non-Hispanic	9,414	792	1.1	7.6 (6.6–8.7)**	7.3 (6.3–8.5)**
Black or African American, non-Hispanic	30,158	4,823	4.9	16.1 (15.3–16.9)**	16.2 (15.4–17.0)**
NH/OPI, non-Hispanic	1,248	172	0.1	15.1 (11.8–19.1)**	14.6 (11.4–18.5)**
White, non-Hispanic (Ref)	295,741	58,598	32.2	20.6 (20.3–20.9)	21.9 (21.5–22.2)
Hispanic or Latino^††^	31,125	5,257	6.3	14.6 (13.8–15.5)**	14.6 (13.7–15.5)**
Multiracial, non-Hispanic	8,135	2,081	0.9	28.5 (26.3–30.8)**	27.9 (25.8–30.1)**
Other, non-Hispanic	3,545	720	0.3	19.3 (17.0–21.9)	19.6 (17.2–22.2)
**Educational attainment**					
Less than high school (Ref)	25,362	5,962	6.6	21.0 (20.0–22.1)	21.2 (20.1–22.3)
High school or equivalent	104,605	19,909	12.8	18.1 (17.6–18.6)**	18.5 (17.9–19.0)**
Technical college or some college	109,074	23,573	16.4	21.0 (20.4–21.5)	21.5 (20.9–22.0)
College degree or higher	151,949	25,182	11.1	14.9 (14.6–15.3)**	15.4 (15.0–15.8)**

**Abbreviations:** AI/AN = American Indian or Alaska Native; NA = not applicable; NH/OPI = Native Hawaiian or other Pacific Islander; Ref = referent group.

* Respondents were classified as having received a diagnosis of depression if they responded “yes” to the question, “Has a doctor, nurse, or other health professional ever told you that you had a depressive disorder, including depression, major depression, dysthymia, or minor depression?”

^†^ Categories might not sum to sample total because of missing responses for some variables.

^§^ Weighted using the statistical weights provided in the Behavioral Risk Factor Surveillance System data set.

^¶^ Except for age groups, estimates were age-standardized to the 2000 projected U.S. Census Bureau population aged ≥18 years using four groups (18–24, 25–44, 45–64, and ≥65 years). https://www.cdc.gov/nchs/data/statnt/statnt20.pdf

** Statistically significant (p<0.05) difference compared with the indicated Ref category.

^††^ Respondents who identified as Hispanic or Latino might be of any race.

Among states, the age-standardized prevalence of depression ranged from 12.7% in Hawaii to 27.5% in West Virginia (median = 19.9%) (Table 2). The 10 states with the highest prevalence were (in descending order) West Virginia, Kentucky, Tennessee, Arkansas, Vermont, Alabama, Louisiana, Washington, Missouri, and Montana. 

**TABLE 2:** State estimates of adults aged ≥18 years self-reporting a lifetime diagnosis of depression* — Behavioral Risk Factor Surveillance System, United States, 2020

Jurisdiction	Sample size	Unweighted no. with depression	Weighted^†^ no. with depression (thousands)	Prevalence^†^ % (95% CI)	Age-standardized prevalence^§^ % (95% CI)
Alabama	5,316	1,252	899	23.5 (22.1–25.0)	23.8 (22.2–25.4)
Alaska	3,649	566	87	15.9 (14.1–17.9)	15.7 (13.9–17.6)
Arizona	10,224	1,867	992	17.4 (16.4–18.4)	17.5 (16.4–18.6)
Arkansas	5,216	1,116	544	23.5 (21.9–25.2)	24.2 (22.4–26.1)
California	4,776	758	4,324	14.1 (12.8–15.5)	13.9 (12.7–15.3)
Colorado	10,131	1,836	842	18.5 (17.6–19.4)	18.5 (17.6–19.4)
Connecticut	8,929	1,649	500	17.7 (16.5–19.0)	18.4 (17.0–19.8)
Delaware	4,010	654	121	15.6 (14.1–17.1)	15.8 (14.3–17.6)
District of Columbia	3,410	621	114	19.8 (18.0–21.7)	19.9 (18.1–21.8)
Florida	11,746	2,038	2,570	14.7 (13.4–16.0)	14.9 (13.5–16.4)
Georgia	9,040	1,618	1,413	17.2 (16.0–18.5)	17.3 (16.0–18.6)
Hawaii	7,735	1,098	141	12.7 (11.8–13.7)	12.7 (11.8–13.8)
Idaho	5,947	1,058	258	18.9 (17.5–20.3)	19.0 (17.6–20.5)
Illinois	3,659	509	1,435	14.7 (13.2–16.4)	15.0 (13.4–16.7)
Indiana	8,435	1,753	1,137	21.9 (20.8–23.0)	22.2 (21.1–23.4)
Iowa	9,606	1,541	422	17.4 (16.5–18.4)	18.1 (17.1–19.1)
Kansas	10,475	1,820	424	19.2 (18.2–20.2)	19.6 (18.5–20.7)
Kentucky	3,918	918	838	24.2 (22.5–26.0)	25.0 (23.2–26.9)
Louisiana	4,722	1,089	829	23.5 (21.9–25.2)	23.8 (22.1–25.6)
Maine	10,935	2,271	241	22.1 (20.8–23.3)	23.1 (21.6–24.6)
Maryland	14,202	2,299	740	15.7 (14.9–16.6)	16.1 (15.1–17.0)
Massachusetts	7,127	1,233	990	17.9 (16.7–19.3)	18.2 (16.8–19.5)
Michigan	7,237	1,385	1,530	19.5 (18.3–20.8)	20.3 (19.0–21.7)
Minnesota	15,781	3,249	864	19.8 (19.0–20.6)	20.2 (19.4–21.0)
Mississippi	6,443	1,224	473	20.9 (19.5–22.3)	21.1 (19.6–22.6)
Missouri	9,162	1,975	1,086	22.8 (21.6–24.0)	23.4 (22.1–24.7)
Montana	6,283	1,310	191	22.6 (21.3–23.9)	23.4 (22.0–24.9)
Nebraska	14,748	2,304	245	16.8 (15.8–17.7)	17.0 (16.0–18.0)
Nevada	2,471	447	429	17.6 (15.6–19.7)	17.5 (15.5–19.6)
New Hampshire	6,411	1,285	238	21.5 (20.0–22.9)	22.5 (20.9–24.2)
New Jersey	11,312	1,832	1,055	15.2 (14.4–16.1)	15.6 (14.7–16.5)
New Mexico	6,984	1,207	284	17.6 (16.1–19.1)	17.8 (16.2–19.5)
New York	14,661	2,559	2,560	16.8 (15.9–17.7)	16.7 (15.9–17.7)
North Carolina	5,817	1,201	1,730	20.8 (19.6–22.2)	20.7 (19.4–22.1)
North Dakota	4,454	708	112	19.2 (17.6–21.0)	19.6 (17.9–21.5)
Ohio	14,592	3,242	2,005	22.0 (21.1–23.0)	22.8 (21.7–23.9)
Oklahoma	5,011	1,129	686	22.9 (21.4–24.4)	23.0 (21.5–24.6)
Oregon	5,380	1,152	712	21.2 (19.9–22.5)	21.4 (20.1–22.8)
Pennsylvania	5,520	1,108	2,056	20.2 (18.9–21.7)	20.9 (19.5–22.5)
Rhode Island	5,348	1,135	180	21.1 (19.5–22.9)	21.4 (19.6–23.3)
South Carolina	3,996	808	876	21.4 (19.8–23.1)	21.5 (19.7–23.3)
South Dakota	6,895	949	108	16.1 (14.1–18.3)	16.4 (14.3–18.8)
Tennessee	4,641	1,152	1,296	24.1 (22.4–25.8)	24.4 (22.6–26.2)
Texas	10,968	2,215	3,881	17.7 (16.4–19.1)	17.5 (16.2–18.9)
Utah	10,861	2,398	537	23.1 (22.1–24.2)	22.7 (21.8–23.7)
Vermont	6,511	1,443	118	23.3 (21.7–25.0)	24.2 (22.4–26.0)
Virginia	9,490	1,670	1,153	17.2 (16.2–18.3)	17.4 (16.3–18.5)
Washington	12,837	3,027	1,412	23.4 (22.5–24.4)	23.5 (22.5–24.5)
West Virginia	5,855	1,530	373	26.4 (25.0–27.8)	27.5 (25.9–29.1)
Wisconsin	5,078	913	904	19.8 (18.3–21.5)	20.5 (18.8–22.3)
Wyoming	4,791	709	81	18.3 (16.6–20.0)	18.9 (17.1–20.8)

* Respondents were classified as having depression if they responded “yes” to the question, “Has a doctor, nurse, or other health professional ever told you that you had a depressive disorder, including depression, major depression, dysthymia, or minor depression?”

^†^ Weighted using the statistical weights provided in the Behavioral Risk Surveillance System data set.

^§^ Age-standardized to the 2000 projected U.S. Census Bureau population aged ≥18 years using four groups (18–24, 25–44, 45–64, and ≥65 years). https://www.cdc.gov/nchs/data/statnt/statnt20.pdf

Among counties, the model-based age-standardized estimates ranged from 10.7% (Aleutians East Borough County, Alaska) to 31.9% (Logan County, West Virginia) (median = 21.8%) (Supplementary Table, https://stacks.cdc.gov/view/cdc/129404); most of the counties with the highest prevalence were in the Appalachian region, the southern Mississippi Valley region, and in Missouri, Oklahoma, and Washington (Figure). Estimates of depression also varied among counties within states. For example, even though all county prevalence estimates in West Virginia were in the highest quartile, estimates in the state by county ranged from 24.5% to 31.9%.

**FIGURE:**
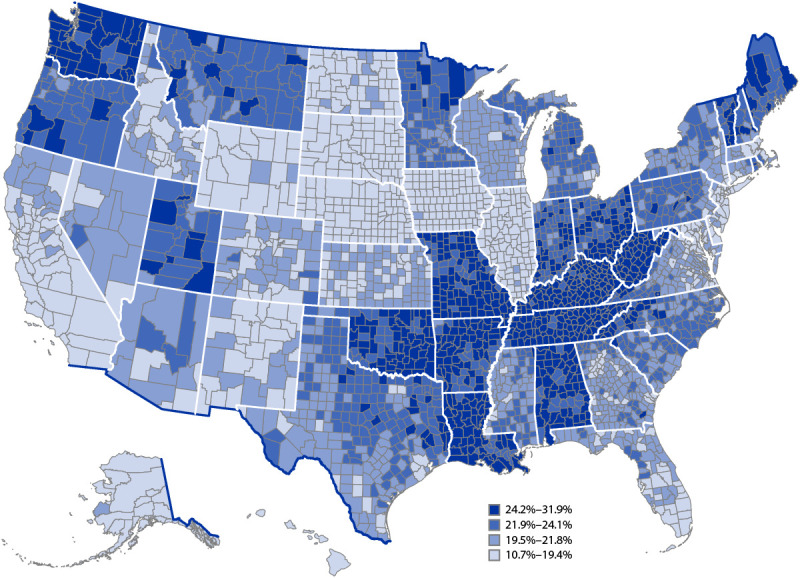
Model-based age-standardized* county estimates of the percentage^†^ of adults aged ≥18 years self-reporting a lifetime diagnosis of depression^§^ — Behavioral Risk Factor Surveillance System, United States, 2020 * Age-standardized to the 2000 projected U.S. Census Bureau population aged ≥18 years using 13 age groups (18–24, 25–29, 30–34, 35–39, 40–44, 45–49, 50–54, 50–59, 60–64, 65–69, 70–74, 75–79, and ≥80 years). https://www.cdc.gov/nchs/data/statnt/statnt20.pdf ^†^ By quartile. ^§^ Respondents were classified as having depression if they responded “yes” to the question, “Has a doctor, nurse, or other health professional ever told you that you had a depressive disorder, including depression, major depression, dysthymia, or minor depression?”

## Discussion

During 2020, approximately one in five U.S. adults reported having ever received a diagnosis of depression by a health care provider, with prevalence of depression higher in women, younger adults, and adults with lower education levels. Previous reports that focused on measures of current depression (e.g., during the previous 2 weeks) rather than lifetime depression showed similar subgroup differences (*2*–*6*), including those observed before and throughout the COVID-19 pandemic (*4*–*6*).

The highest prevalence of having ever been diagnosed with depression by a health care professional was found among young adults (aged 18–24 years). Data from the National Survey on Drug Use and Health show that during 2015 to 2019, previous-year depression increased most rapidly among adolescents (aged 12–17 years) and young adults (aged 18–25 years) (*4*). Depression can affect people differently by age,^†††^ and the American Psychological Association provides clinical practice guidelines and decision aids for the treatment of depression by age group, including children and adolescents, adults, and older adults.^§§§^ Health promoting behaviors, including physical activity, quality sleep, and good nutrition, can help to manage symptoms of depression and support positive mental health across the lifespan (*7*).^¶¶¶^

There was considerable geographic variation in the prevalence of depression, with the highest state and county estimates of depression observed along the Appalachian and southern Mississippi Valley regions. Depression is a comorbidity for many chronic diseases, including diabetes, arthritis, and cardiovascular diseases (*8*). These diseases also occur in higher concentrations in states within the Appalachian region,**** suggesting that geographic variation in the prevalence of depression might partially reflect patterns of other chronic diseases. The variation in depression might also reflect the influence of social determinants of health^††††^ in counties and states, including economic status and differences in access to health care. For example, adults in the Appalachian region tend to have lower incomes, higher poverty rates, and lower education levels, all of which can negatively affect health and well-being.^§§§§^ The model-based county-level estimates provided in this report offer a starting point for identifying geographic disparities in depression. Incorporating additional neighborhood-level data and context can help guide local public health practitioners in the development and implementation of effective and targeted efforts to address mental health in their communities.

Population-level efforts to address prevention, treatment, and management of depression include tailored and targeted programs to address demographic and geographic disparities. CDC provides information about mental health resources and programs, including those focused on specific populations (e.g., children, older adults, and those with chronic conditions). Depression often co-occurs with other health conditions, and chronic disease self-management programs also help persons with chronic conditions manage their disease and improve their mental health.^¶¶¶¶^ CDC’s How Right Now***** is a communications campaign designed to promote and strengthen the emotional well-being and resilience of persons disproportionally affected by mental health challenges. The campaign offers evidence-based information and resources to address the emotional health needs of adults (*9*).

In addition, CPSTF provides communities with a list of recommended interventions to improve mental health or address mental illness.^†††††^ Examples of recommended interventions include collaborative care for the management of depressive disorders, mental health benefits legislation, school-based cognitive behavioral therapy programs to reduce depression and anxiety symptoms (targeted and universal), and depression care management among older adults (clinic- and home-based). SAMHSA’s Evidence-Based Practices Resource Center also provides communities, clinicians, policymakers and others with the information and tools to incorporate evidence-based practices into their communities or clinical settings.^§§§§§^

The findings in this report are subject to at least three limitations. First, BRFSS collects self-reported data about whether the respondent has ever received a diagnosis of depression from a health care professional and these data are susceptible to recall, nonresponse, cultural and social desirability, and other reporting biases (*10*). Differences in reporting could be attributed to sex, cultural, and generational differences, and a person’s willingness to discuss symptoms with a health care provider, as well as their access to a provider. Second, data are collected via a landline and cell phone survey that excludes institutionalized populations or those who cannot be reached via this method, which might affect the representativeness of the samples. Finally, county estimates of prevalence might be imprecise because the multilevel regression modeling approach uses the U.S. Census Bureau Vintage 2020 county population estimates, which are estimates of the population rather than census counts.

This report provides current estimates of national, state-level, and county-level prevalence of adults reporting a lifetime diagnosis of depression. These estimates can help decision-makers guide resource allocation to areas where the need is greatest, which might include consideration of evidence-based interventions and practices such as those recommended by CPSTF and SAMHSA.

**Summary**

**What is already known about this topic?**

Depression is a major cause of morbidity and mortality in the United States.

**What is added by this report?**

During 2020, 18.4% of U.S. adults reported having ever been diagnosed with depression; state-level age-standardized estimates ranged from 12.7% in Hawaii to 27.5% in West Virginia. Model-based age-standardized county-level prevalence estimates ranged from 10.7% to 31.9%, and there was considerable state-level and county-level variability.

**What are implications for public health practice?**

Decision-makers can use these estimates to guide resource allocation to areas where the need is greatest, possibly by implementing practices such as those recommended by The Guide to Community Preventive Services Task Force and the Substance Abuse and Mental Health Services Administration.

## Footnotes

* The Appalachian region includes all of West Virginia and parts of Alabama, Georgia, Kentucky, Maryland, Mississippi, New York, North Carolina, Ohio, Pennsylvania, South Carolina, Tennessee, and Virginia (https://www.arc.gov/about-the-appalachian-region/); Alabama, Kentucky, Tennessee, and West Virginia were among the 10 states with the highest prevalence.

† The southern Mississippi Valley region includes parts of Arkansas, Louisiana, Mississippi, and Tennessee (https://www.usace.army.mil/Missions/Locations/); Arkansas, Louisiana, and Tennessee were among the 10 states with the highest prevalence.

§
https://www.cdc.gov/brfss/about/index.htm

¶
https://www.cdc.gov/brfss/annual_data/annual_2020.html

** The method for the small area estimate approach is used for the PLACES project conducted by CDC, which provides estimates for 29 chronic disease measures at county, place (incorporated and census-designated), census tract, and zip code tabulation area levels. The method does not include territories as part of the 3,143 counties. https://www.cdc.gov/places/methodology/index.html

††
https://www.census.gov/programs-surveys/acs/

§§
https://www.census.gov/programs-surveys/popest/technical-documentation/research/evaluation-estimates/2020-evaluation-estimates/2010s-county-detail.html

¶¶
https://www.cdc.gov/nchs/data/statnt/statnt20.pdf

*** 45 C.F.R. part 46.102(1)(2), 21 C.F.R. part 56; 42 U.S.C. Sect. 241(d); 5 U.S.C. Sect. 552a; 44 U.S.C. Sect. 3501 et seq.

†††
https://www.nimh.nih.gov/health/publications/depression

§§§
https://www.apa.org/depression-guideline

¶¶¶
https://www.cdc.gov/mentalhealth/

****
https://www.arc.gov/wp-content/uploads/2020/06/Health_Disparities_in_Appalachia_August_2017.pdf

††††
https://www.cdc.gov/chronicdisease/programs-impact/sdoh.htm

§§§§
https://health.gov/healthypeople/priority-areas/social-determinants-health

¶¶¶¶
https://www.cdc.gov/arthritis/interventions/self_manage.htm

*****
https://www.cdc.gov/howrightnow/

†††††
https://www.thecommunityguide.org/pages/task-force-findings-mental-health.html

§§§§§
https://www.samhsa.gov/resource-search/ebp
